# Distinct prostate cancer-related mRNA cargo in extracellular vesicle subsets from prostate cell lines

**DOI:** 10.1186/s12885-017-3087-x

**Published:** 2017-02-01

**Authors:** Elisa Lázaro-Ibáñez, Taral R. Lunavat, Su Chul Jang, Carmen Escobedo-Lucea, Jorge Oliver-De La Cruz, Pia Siljander, Jan Lötvall, Marjo Yliperttula

**Affiliations:** 1 Division of Pharmaceutical Biosciences, Centre for Drug Research, Faculty of Pharmacy, University of Helsinki, Helsinki, 00014 Finland; 2 Krefting Research Center, Department of Internal Medicine and Clinical Nutrition, University of Gothenburg, Gothenburg, 40530 Sweden; 3 Institute for Advanced Biomedical Engineering, Tokyo Women´s Medical University (TWINS), Tokyo, 162 8666 Japan; 4 Center for Translational Medicine, International Clinical Research Center, St. Anne’s University Hospital, Brno, 65691 Czech Republic; 5 Division of Biochemistry and Biotechnology, Department of Biosciences, University of Helsinki, Helsinki, 00014 Finland; 6 Division of Pharmaceutical Sciences, Faculty of Pharmacy, University of Padova, Padova, 35131 Italy

**Keywords:** mRNA, Extracellular vesicles, Microvesicles, Exosomes, Prostate cancer

## Abstract

**Background:**

Multiple types of extracellular vesicles (EVs), including microvesicles (MVs) and exosomes (EXOs), are released by all cells constituting part of the cellular EV secretome. The bioactive cargo of EVs can be shuffled between cells and consists of lipids, metabolites, proteins, and nucleic acids, including multiple RNA species from non-coding RNAs to messenger RNAs (mRNAs). In this study, we hypothesized that the mRNA cargo of EVs could differ based on the EV cellular origin and subpopulation analyzed.

**Methods:**

We isolated MVs and EXOs from PC-3 and LNCaP prostate cancer cells by differential centrifugation and compared them to EVs derived from the benign PNT2 prostate cells. The relative mRNA levels of 84 prostate cancer-related genes were investigated and validated using quantitative reverse transcription PCR arrays.

**Results:**

Based on the mRNA abundance, MVs rather than EXOs were enriched in the analyzed transcripts, providing a snapshot of the tumor transcriptome. LNCaP MVs specifically contained significantly increased mRNA levels of NK3 Homeobox 1 (*NKX3-1)*, transmembrane protease serine 2 (*TMPRSS2),* and tumor protein 53 (*TP53)* genes, whereas PC-3 MVs carried increased mRNA levels of several genes including, caveolin-2 (*CAV2)*, glutathione S-transferase pi 1 (*GSTP1)*, pescadillo ribosomal biogenesis factor 1 (*PES1*), calmodulin regulated spectrin associated protein 1 (*CAMSAP1*), zinc-finger protein 185 (*ZNF185)*, and others compared to PNT2 MVs. Additionally, ETS variant 1 (*ETV1)* and fatty acid synthase (*FASN)* mRNAs identified in LNCaP- and PC-3- derived MVs highly correlated with prostate cancer progression.

**Conclusions:**

Our study provides new understandings of the variability of the mRNA cargo of MVs and EXOs from different cell lines despite same cancer origin, which is essential to better understand the the proportion of the cell transcriptome that can be detected within EVs and to evaluate their role in disease diagnosis.

**Electronic supplementary material:**

The online version of this article (doi:10.1186/s12885-017-3087-x) contains supplementary material, which is available to authorized users.

## Background

Extracellular vesicles (EVs) are membrane-derived particles released under normal conditions by many cell types to eliminate non-desired cell components and to share information between cells and their environment [1, 2]. EVs are considered to play important roles in cell-to-cell communication, contributing to changes in the recipient cell phenotypes by influencing their functions, which is of special relevance for instance in the hallmarks of cancer [3, 4]. Cells release a diverse mixture of EVs that can be considered as the cellular EV secretome [5–7]. Most notable subpopulations of EVs are microvesicles (MVs) and exosomes (EXOs), both released by viable cells under normal physiological conditions. Current state of the art classification considers MVs to have a diameter of 100–1,000 nm, and being primarily derived from the cell surface by budding of the plasma membrane, whereas EXOs are smaller, 30–150 nm, and are formed within the multivesicular bodies which liberate EXOs to the extracellular space by fusion with the plasma membrane [8]. Importantly, EVs contain a broad variety of signaling molecules that reflect the composition of their originator cells including lipids, proteins, metabolites, sugars, and particularly nucleic acids [2, 8]. These molecules can be functionally delivered mediating biological activities and functional changes in the recipient cells [9–12]. Interestingly, the EV cargo only partly reflects the cells of origin, and especially EXOs have been shown to carry different RNA species compared to the producing cells [9, 10, 13–15].

Several RNA molecules, including messenger RNAs (mRNAs) and non-coding RNAs (ncRNAs), particularly microRNAs (miRNAs) but also transfer RNAs and other ncRNAs, have been investigated from EVs of different origins [15–17]. MVs and EXOs share functional features, and to a large extent, molecular composition. However, they are still distinct subpopulations of EVs that are likely to mediate different actions. As the most common methods of vesicle isolation do not allow separation of EV subsets, most EV studies have not compared MVs and EXOs, and hence a very limited number of reports have so far compared the cargo differences of the EV subtypes [5, 6, 18–20].

We hypothesized that the mRNA content of EVs differs based on the EV subpopulation analyzed and the cellular origin of the EVs. Moreover, we focused on the comparison of the mRNA cargo of MVs and EXOs from different prostate cancer (LNCaP and PC-3) and benign (PNT2) cells, since such comparison has so far not been performed. Quantitative reverse transcription PCR (RT-qPCR) mRNA arrays were used to characterize and validate the mRNA signatures of 84 prostate cancer (PCa) related genes in the EVs. We analyzed mRNAs known to be related to PCa, as such data could provide new insights for understanding the differential mRNA content of EV subpopulations which may be of future clinical use.

## Methods

### Cell culture

LNCaP and PC-3 (ATCC, Manassas, VA, USA) were grown in RPMI 1640 and DMEM/F12 media (Sigma-Aldrich, St Louis, MO, USA), respectively. Both media were supplemented with 10% (v/v) EV-depleted FBS (Sigma-Aldrich). The FBS was EV-depleted by ultracentrifugation at 118,000 x *g*_*avg*_ for 18 h using a type 45 Ti rotor k-factor 178.6 (Beckmann Coulter, Brea, CA, USA), followed by filtration through a 0.22 μm filter (Merck Millipore, Billerica, Massachusetts, USA). Immortalized human benign prostate epithelial cells PNT2 (ECACC, Sigma-Aldrich) were grown in serum-free defined keratinocyte media, supplemented with bovine pituitary extract and human recombinant epidermal growth factor (Life Technologies, Carlsbad, CA, USA). All media were supplemented with 100 IU/mL of penicillin and 100 μg/mL streptomycin (HyClone, Logan, UT, USA). Cells were cultivated at 37 °C and 5% CO_2_. Cell viability was measured by Trypan Blue solution (Sigma-Aldrich). Cells were routinely checked for mycoplasma contamination using the MycoAlert™ PLUS (Lonza Walkersville, MD, USA).

### Extracellular vesicle isolation

Three hundred mL of cell cultured conditioned media was harvested from LNCaP, PC-3, and PNT2 cells at 80% confluence, and centrifuged at 1,000 x *g* at 4 °C for 10 min to remove remaining cells and cellular debris. The remaining supernatant was centrifuged at 2,500 x *g* for 25 min at 4 °C to pellet larger vesicles such apoptotic bodies. The supernatant was transferred to new tubes and centrifuged at 20,000 x *g*_*avg*_ for 25 min at 4 °C to pellet the MV-enriched fractions. The final supernatant was ultracentrifuged at 110,000 x *g*_*avg*_, for 2 h using an Optima XE 90 ultracentrifuge, 45 Ti rotor and polyallomer centrifuge tubes (Beckman Coulter) k-factor 191.3, to pellet the EXO-enriched fractions. All vesicle pellets were resuspended in PBS or lysis buffer depending on the down-stream analysis and stored at -80 °C.

### Transmission electron microscopy

MV and EXO samples (6 μL) were incubated onto glow discharged 200 mesh formvar copper grids (Electron Microscopy Science) for 2 min at 4 °C. The grids were washed and blotted dry with filter paper. Next, the grids were negatively stained with 2% aqueous uranyl acetate (Sigma-Aldrich), washed with distilled water and dried in darkness. The grids were visualized using a transmission electron microscope (FEI Tecnai Spirit G2) at 80 kV. Images were taken by a digital camera (Soft Image System, Morada).

### Western blot analysis

EVs and cells were lysed with RIPA buffer (Pierce, Thermo Scientific, Rockford, IL, USA) and sonicated three times for five minutes with intermittent vortexing in between. Protein concentration was determined by using the BCA Protein assay following manufacturer´s recommendations (Pierce, Thermo Scientific). Then, 30 μg of protein of cellular lysates and EVs were loaded on a 10% polyacrylamide gel and transferred onto a nitrocellulose membrane (Bio-Rad laboratories, Hercules, CA, USA). Membranes were blocked with 5% Blotting-Grade Blocker Non-Fat Dry Milk (Bio-Rad Laboratories) in Tris-buffer saline (TBS) for 1 h. Membranes were incubated with the following primary antibodies dissolved in 0.25% Blotting-Grade Blocker Non-Fat Dry Milk in TBS-0.5% Tween-20 (TBST) against: calnexin (1:1000; clone H-70), CD81 (1:800; clone H-121;), and flotillin-1 (1:1000; clone H-104) all from Santa Cruz Biotechnology (Santa Cruz, CA, USA) at 4 °C overnight. The membranes were washed three times with TBST, and incubated for 1 h at room temperature with the secondary antibody (1:10,000) ECL donkey anti-rabbit IgG horseradish peroxidase-linked F(ab’)_2_fragment (GE Healthcare, Buckinghamshire, UK), diluted in 0.25% Milk in TBST. The membranes were washed three times with TBST and analyzed with ECL Prime Western Blotting Detection (GE Healthcare) and a VersaDoc 4000 MP (Bio-Rad Laboratories).

### RNA extraction and profiling

Total RNA from cells and EV subpopulations was isolated using the miRNAeasy micro kit (Qiagen, Hilden, Germany), according to the manufacturer´s instructions. RNA samples were eluted in 14 μL of RNAse-free water, aliquoted, and stored at -80 °C. The quality and sized of the isolated RNA was measured by capillary electrophoresis using an Agilent Bioanalyzer 2100 (Agilent Technologies, Santa Clara, CA, USA). RNA from each sample was denatured at 72 °C for 2 min and loaded into RNA 6000 Nano and Pico total RNA kits (Agilent Technologies) to analyze RNA profile and concentration. The RNA concentration was used as a normalizing loading factor for all EV samples.

### Reverse transcription and pre-amplification

RNA samples were subjected to genomic DNA removal; cDNA synthesis, and instant preamplification using the RT^2^ PCR System PreAMP and Human Prostate Cancer Pathway mix (Qiagen, Hilden, Germany), following the manufacturer’s recommendations. Next, pre-amplified cDNA was quantified by using Bioanalyzer 2100 and Nanodrop 1000 (Thermo Scientific) and input to the PCR arrays was normalized to 100 ng/μL for all samples.

### Reverse transcription quantitative PCR (RT-qPCR)

The PAHS-135Z Human Prostate Cancer Pathway RT^2^ Profiler PCR Arrays (SABiosciences, Qiagen) were used to analyze all vesicle samples, following the protocol´s instructions. For each sample type, three assays were carried out as independent biological replicates. In summary, 100 ng/μL of pre-amplified cDNA from LNCaP-, PC-3- and PNT2- derived MVs and EXOs were mixed with the RT^2^ SYBR Green Mastermix and RNAse-free water. Aliquots of the PCR component mix (25 μL) were distributed across the array. The plates were sealed, centrifuged 1 min at 1,500 x *g* to remove air bubbles, and run in a CFX96 thermocycler (Bio-Rad). The cycling conditions were as follows: 95 °C for 10 min; 40 cycles (95 °C for 15 sec, 60 °C for 1:00 min). The Ramp rate between the 95 °C to 60 °C step was 1 °C/sec and the same threshold was used across the arrays. Each array contained inter-plate and reverse transcription calibrators as well as a gDNA contamination control.

### mRNA data analysis

Raw cycle threshold (Ct) values were exported and analyzed by using the PCR Array Data Analysis Software version 3.5, provided by SABiosciences. Gene mRNA level was related to the mean mRNA levels of all the genes present across the samples. Only Ct values < 35 were included in the analysis. Calculations of relative expression were performed with the 2^−ΔΔCT^ method [21]. Student’s *t*-test was used to calculate the *P* values of the replicate 2^−ΔCt^ values for each gene in the control and sample groups. *P* values of less than 0.05 were considered statistically significant. A fold-change threshold of 10 was used for the stringent analysis. Results are shown as the mean ± SEM of three samples for each condition in relation to the mean ± SEM of three control samples for each group. All statistical analyses were performed using the statistical software package, GraphPad Prism 7.0 (GraphPad Software, Inc., San Diego, CA, USA).

## Results

### Differential RNA profiles in the subpopulations of EVs from PCa and non-cancerous cells

**Fig. 1:**
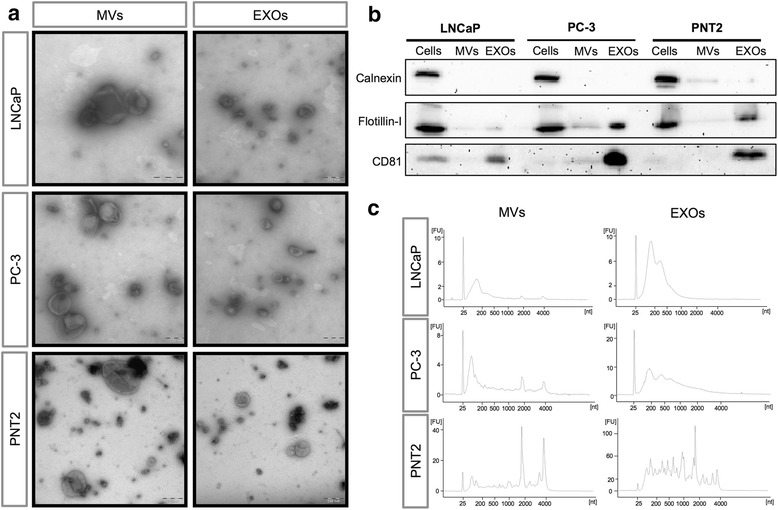
Characterization of the EV subpopulations isolated from prostate cell lines. **a** Representative electron microscopy pictures of microvesicles (MVs) and exosomes (EXOs) isolated from LNCaP, PC-3 and PNT2 cells by differential centrifugation. Scale bars represent 200 nm. **b** Presence of calnexin (an endoplasmic reticulum marker), flotillin-I, and CD81 (EV markers) analyzed by Western blotting. An equal protein amount of 30 μg was loaded of all the samples. **c** RNA profiles of MVs and EXOs isolated from LNCaP, PC-3, and PNT2 cells after total RNA extraction. Profiles analyzed by capillary electrophoresis with the Agilent RNA 6000 Nano and Pico Total RNA kits. Representative electropherograms showing in the y-axis fluorescence units (FU) and in the x-axis the nucleotide length (nt) of the RNA. Peaks at 25 nt represent internal standards and peaks at 2,000 nt and 4000 nt represent ribosomal RNA 18 and 28 subunits, respectively. The panels A-C are representative of 3 independent experiments showing the same trends of vesicle morphology, size, and presence of the selected markers

Using differential centrifugation, we isolated both MVs and EXOs from two common PCa cell lines (LNCaP and PC-3), and a benign prostate epithelial cell line (PNT2). Transmission electron microscopy characterization showed that MVs were more heterogeneous in size and morphology than EXOs, ranging in sizes from > 200 nm, while the EXOs varied between 30–150 nm (Fig. 1a). Additionally, no evident differences were observed in EV morphology among the three cell lines analyzed. The membrane proteins CD81 and flotillin-1 (common EV markers) were detected in both EV subpopulations by Western blotting with a higher enrichment in the EXO fractions compared to MVs. The endoplasmic reticulum marker calnexin was highly enriched in cell samples, compared to EV fractions (Fig. 1b).

To determine whether EVs isolated from the PCa and non-cancerous cell lines had distinct RNA profiles, we analyzed the total RNA content and length from their MVs and EXOs using a Bioanalyzer 2100 instrument (Fig. 1c). MVs from all three samples had clear and distinct peaks at around 2,000 and 4,000 nucleotides (nt) which represent the 18S and 28S ribosomal RNA (rRNA) respectively, with relatively moderate levels of small RNAs. In contrast, EXOs were primarily enriched in small RNAs but also contained larger RNA molecules, especially in the PNT2 EXOs RNA profile. MV and EXO RNAs from both PCa cell lines had similar profiles and size distributions. On the other hand, MVs and EXOs derived from non-cancerous cells had very similar RNA profiles between subpopulations but different RNA profiles compared with the cancer-derived EVs, suggesting that the RNA content of EVs from distinct cellular origins significantly varies.

### MVs and EXOs from different cell lines have unique mRNA signatures

**Fig. 2:**
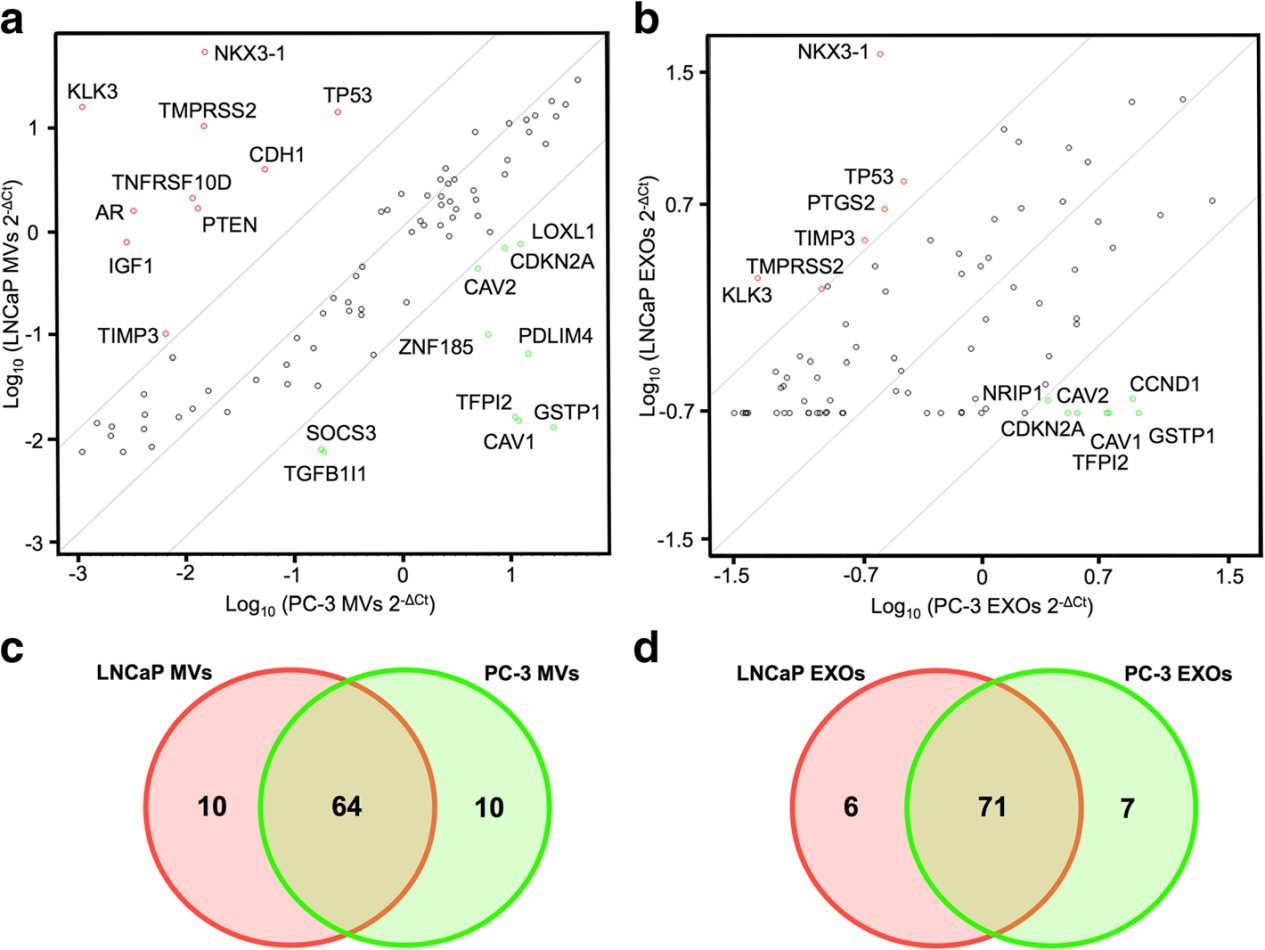
Prostate cancer-derived MVs and EXOs have unique mRNA signature resembling their origin. Scatter plots depicting a log transformation of the relative mRNA level of each gene (2^-ΔCt^) between: (**a**) LNCaP MVs (y-axis) and PC-3 MVs (x-axis) and (**b**) LNCaP EXOs (y-axis) and PC-3 EXOs (x-axis). The gray lines indicate a boundary of 10. Genes at prominent coordinates are annotated. Data are representative of three independent experiments per group. Fold-change cut-off =10. (**c**, **d**) Venn diagrams representing common and unique mRNAs of the genes detected in LNCaP and PC3 MVs and LNCaP and PC-3 EXOs respectively

As the RNA profiles found in the EV subpopulations seem to represent a diverse selection of RNA species, we decided to focus further analyses on the mRNA content of EVs. For that purpose, we used a human PCa pathway qPCR array approach, which covers 84 mRNA transcripts of genes known to be involved in PCa (Fig. 2 and Additional file 1: Figure S1). First, we determined the percentage of genes detected in the different groups by the presence of specific mRNAs. Close to 100% of these mRNAs were detected in MVs from both LNCaP and PC-3 cells, whereas less than 50% of the mRNAs studied were identified in MVs from the PNT2 cells. Additionally, the abundance of the different transcripts analyzed was more variable in EXOs than in MVs among the different donor cells, as observed by the Ct values (Additional file 1: Figure S1). Overall, the Ct values obtained from the analysis were lower in the MVs compared to the EXOs in all samples, suggesting an enrichment of these mRNAs in MVs.

To examine whether distinct mRNA patterns were observed in EVs derived from different PCa cells, we compared the transcripts present in LNCaP and PC-3 cell-derived MVs and EXOs (Fig. 2). The differences in mRNAs abundances are shown as a scatter plots in Fig. 2a, b, with the cut-off lines being 10-fold change. We used this strict limit to focus on transcripts with obvious differences among the samples. The mRNA transcripts from ten genes were shown to be unique for LNCaP MVs, and ten different mRNA transcripts were also specific for PC-3 MVs (Fig. 2c). On the other hand, six transcripts were unique for LNCaP EXOs, and seven other mRNA transcripts were exclusive of PC-3 EXOs (Fig. 2d).

Among the commonly found mRNAs that were differentially present in both MVs and EXOs for each cell line, LNCaP samples showed significant increases of kallikrein-related peptidase 3 (*KLK3)* (also known as prostate-specific antigen: PSA) related to PCa neoplastic growth and metastasis [22]; NK3 Homeobox 1 (*NKX3-1*), an androgen-regulated transcription factor indicating poor prognosis [23]; the transmembrane protease serine 2 (*TMPRSS2*), a gene which is up-regulated by androgenic hormones [23]; the tissue inhibitor of metalloproteinase-3 gene (*TIMP3*), a tumor suppressor gene frequently down-regulated in PCa [24]; and tumor protein 53 (*TP53)*, the well-known tumor suppressor gene. In contrast, for PC-3 MVs and EXOs a common increase in the mRNA levels of several genes was found, including caveolin-1 (*CAV1*), reportedly overexpressed in PCa and associated with disease progression [25, 26]; caveolin-2 (*CAV2)*, a gene involved in cell cycle [27]; glutathione S-transferase pi 1 (*GSTP1*) and cyclin-dependent kinase inhibitor 2A (*CDKN2A*), both genes showing differentially methylated promoters and involved in cancer progression [28, 29]; and tissue factor pathway inhibitor 2 (*TFPI2*), a gene usually down-regulated in PCa cells [30]. These differences between EVs based on their source of origin defined their specific mRNA signature.

### mRNA transcripts associated with PCa can be variably detected in EV subpopulations

**Fig. 3:**
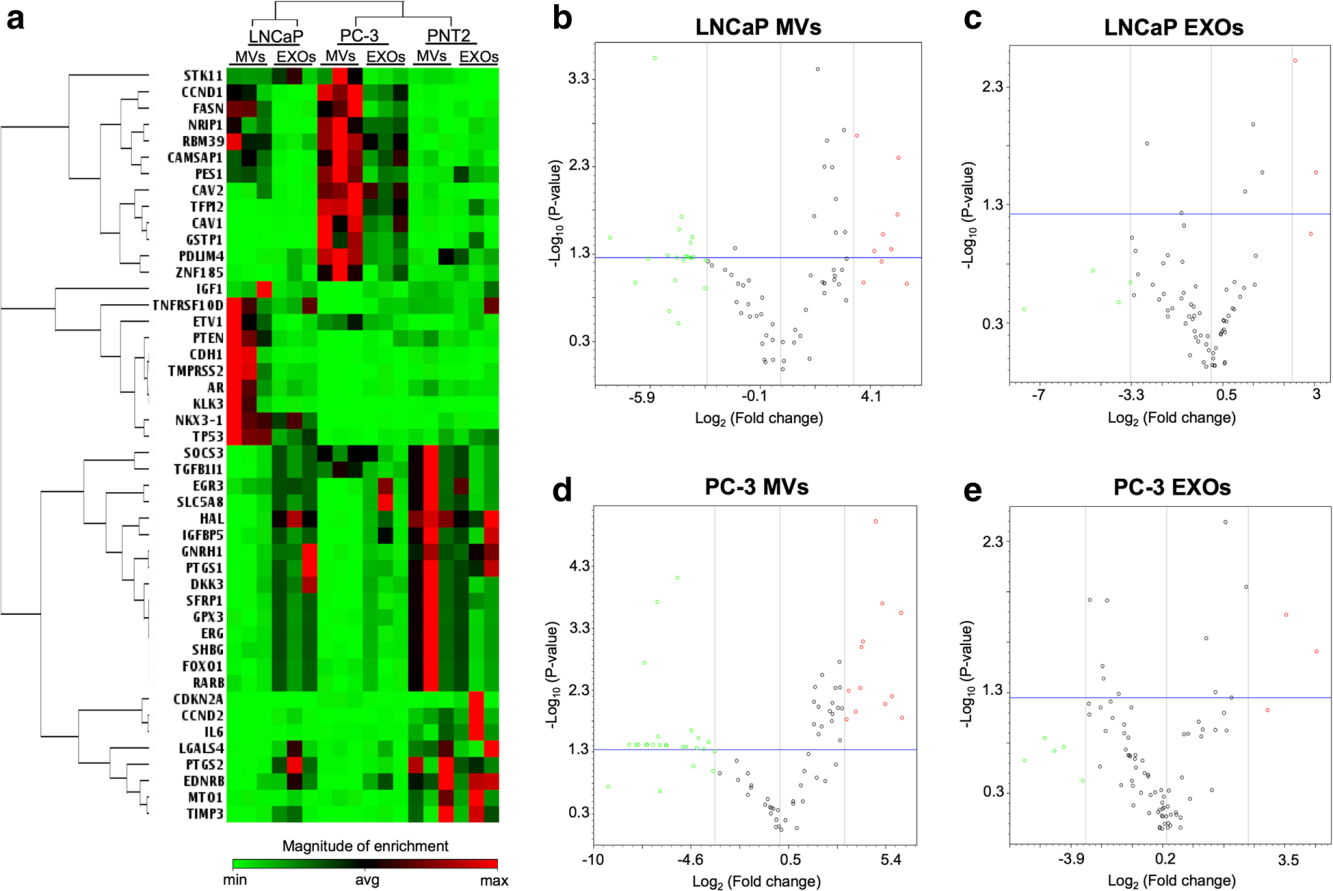
Comparison of the differences in the mRNA levels associated with MVs and EXOs. **a** Non-supervised hierarchical clustering indicating normalized enrichment of the mRNA levels of the 46 most significant genes detected in the LNCaP and PC-3 MVs and EXOs in comparison with the PNT2 MVs or PNT2 EXOs. Volcano plots indicating changes in mRNA levels between: (**b**) LNCaP MVs *vs* PNT2 MVs; (**c**) LNCaP EXOs *vs* PNT2 EXOs; (**d**) PC-3 MVs *vs* PNT2 MVs; and (**e**) PC-3 EXOs *vs* PNT2 EXOs. Data is representative of three independent experiments per group. Data is reported as x-axis = log_2_ (fold change of sample/Ctrl), y-axis = negative log_10_ (*P* value). The horizontal blue line indicates a *P* value of 0.05. The vertical grey line indicates an absolute fold-change of 10. Red and green dots indicate increased or decreased mRNA levels of the genes of study in the MVs and EXOs in comparison to the PNT2 MVs and EXOs

A hierarchical clustering analysis of the most significantly differing mRNAs in MVs and EXOs across the groups was performed in order to evidence the abundance pattern of each sample (Fig. 3a). The PCa cell-derived EV subpopulations had more similarities with each other compared with the non-cancerous EVs, and the obtained clustering pattern reflected their differences of cellular origin (cancerous *vs* non-cancerous cells) (Fig. 3a). Next, the relative mRNA levels between MVs and EXOs in comparison with their respective EV control from the benign cells were examined. Volcano plots showed significances (as a negative log *P* values) *vs* means of differential fold changes for the comparisons of LNCaP MVs *vs* PNT2 MVs (Fig. 3b), LNCaP EXOs *vs* PNT2 EXOs (Fig. 3c), PC-3 MVs *vs* PNT2 MVs (Fig. 3d), and PC-3 EXOs *vs* PNT2 EXOs (Fig. 3e). Using a cut-off threshold of 10-fold change and a *P* value of 0.05, the number of genes classified based on their relative abundance on the study groups compared with the control group was similar [LNCaP MVs: 21/9, LNCaP EXOs: 4/3, PC-3 MVs: 25/12, PC-3 EXOs; 5/3 (up/down)].

Comparison of MVs and EXOs showed that MVs were enriched in the studied mRNAs compared to the EXOs for both cell lines (Fig. 3b-e). Furthermore, the statistically significant mRNA transcripts in EXOs samples were also present in the MVs. In view of both the RNA concentration and the transcript enrichment, MVs were selected to further study the differences in the mRNA levels known to be associated with PCa.

### MVs contained differential PCa-associated mRNAs

**Fig. 4:**
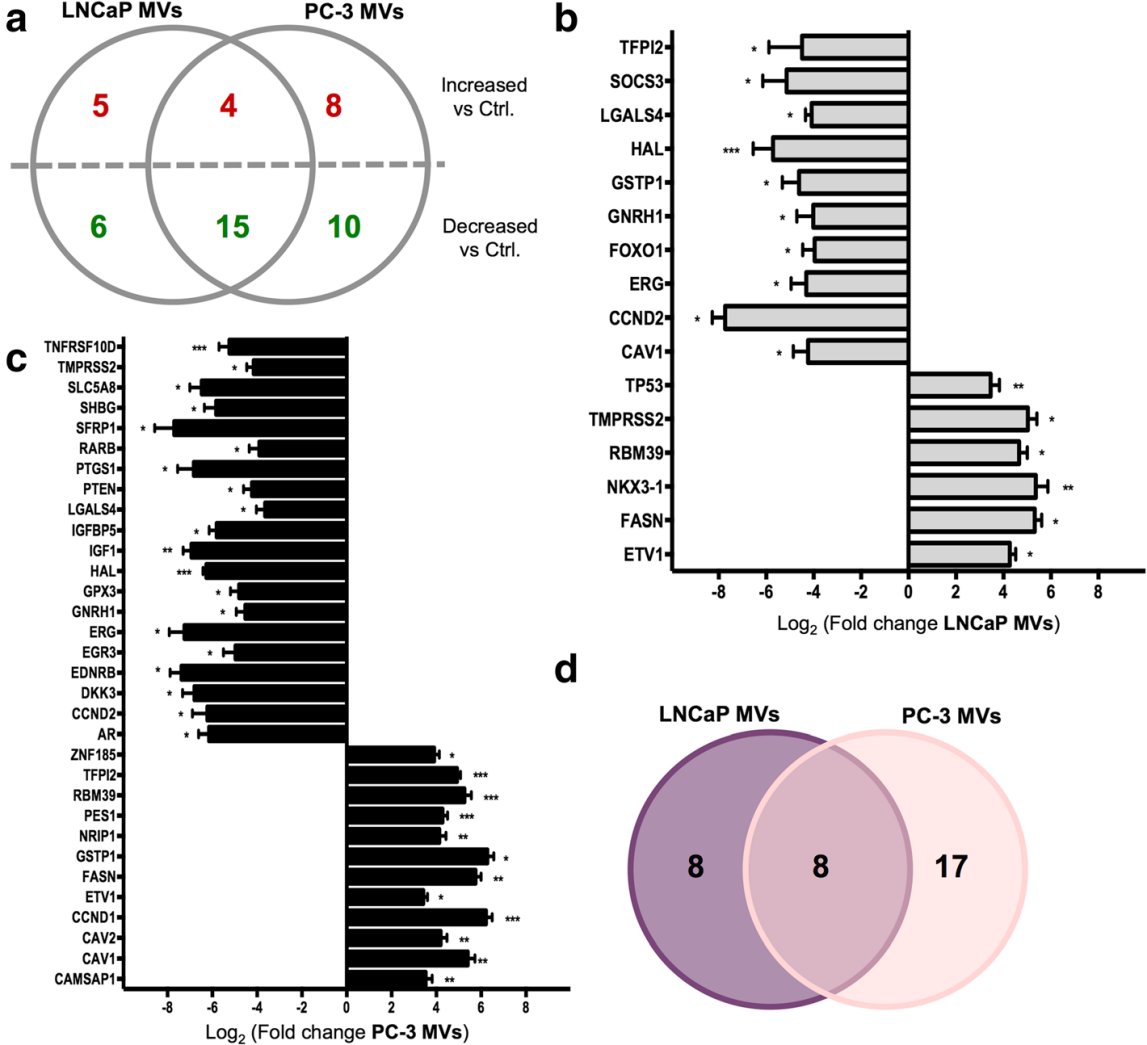
Prostate cancer associated mRNA analysis in MVs. **a** Venn diagram representing increased (*red*) and decreased (*green*) mRNA levels of prostate cancer (PCa) pathway genes in the LNCaP- and PC-3 MVs *vs* the PNT2 MVs (Ctrl). RT-qPCR data showing the statistically significant fold changes of particular genes in (**b**) LNCaP MVs *vs* Ctrl (**c**) PC-3 MVs *vs* Ctrl. Data are reported as the log_2_ of the fold change relative to the Ctrl. Fold-change cut-off =10. Bars represent the mean ± SEM of three independent experiments per group. *P <0.05, **P <0.01, ***P <0.001. **d** Venn diagram representing a summary the statistically significant mRNAs of the analyzed genes after comparison with the Ctrl, exclusively present in LNCaP MVs and PC-3 MVs. The mRNAs that were significantly common for both samples are also represented

To compare the studied MVs from the different origins in depth, we examined the differential mRNA abundance across all the MV samples (Fig. 4). For both LNCaP and PC-3 MVs, there was a 55.8% overlap in mRNA transcripts when compared to PNT2 MVs (Fig. 4a). Additionally, mRNAs were classified as enriched either exclusively in the LNCaP MVs or PC-3 MVs, or to be common for both the PCa vesicle types (Additional file 2: Table S1, Additional file 3: Table S2 and Additional file 4: Table S3). The statistically significant mRNA transcripts for LNCaP MVs (Fig. 4b) and PC-3 MVs (Fig. 4c) are presented. The analysis of the fold differences between groups showed that the mRNA abundance of 30 genes in the LNCaP MVs (16 statistically significant) and 37 in the PC-3 MVs (32 statistically significant) was different from the control PNT2 MVs. For LNCaP MVs, mRNA levels of ETS variant 1 (*ETV1)*, fatty acid synthase (*FASN)*, *NKX3-1*, RNA-binding protein 39 (*RBM39)*, *TMPRSS2*, and *TP53* genes were increased, while mRNA levels of *CAV1*, cyclin D2 (*CCND2)*, ETS-related gene (*ERG)*, forkhead box protein O1 (*FOXO1)*, gonadotropin releasing hormone 1 (*GNRH1)*, *GSTP1*, histidine ammonia-lyase (*HAL)*, lectin, galactoside binding soluble 4 (*LGALS4)*, suppressor of cytokine signaling 3 (*SOCS3)*, and tissue factor pathway inhibitor 2 (*TFIPI2)* genes were decreased when compared to PNT2 MVs (Fig. 4b). From PC-3 MVs, increased mRNA levels of calmodulin regulated spectrin associated protein 1 (*CAMSAP1)*, *CAV1*, *CAV2*, cyclin D1 (*CCND1)*, *ETV1*, *FASN*, *GSTP1*, nuclear receptor interacting protein 1 (*NRIP1)*, pescadillo ribosomal biogenesis factor 1 (*PES1)*, *RBM39*, *TFPI2*, *ZNF185* were detected, whereas mRNA levels of androgen receptor (*AR)*, *CCND2*, dickkopf WNT signaling pathway inhibitor 3 (*DKK3)*, endothelin receptor type B (*EDNRB)*, early growth response 3 (*EGR3)*, *GNRH1*, glutathione peroxidase 3 (*GPX3)*, *HAL*, insulin like growth factor 1 (*IGF1)*, insulin like growth factor binding protein 5 (*IGFBP5)*, *LGALS4*, phosphatase and tensin homolog (*PTEN)*, prostaglandin G/H synthase and cyclooxygenase (*PTGS1)*, retinoic acid receptor beta (*RARB)*, secreted frizzled-related protein 1 (*SFRP1)*, sex hormone binding globulin (*SHBG)*, solute carrier family 5 member 8 (*SLC5A8)*, *TMPRSS2*, tumor necrosis factor receptor superfamily member 10d (*TNFRSF10D)* were decreased when compared to PNT2 MVs (Fig. 4c). Overall, when analyzing the statistically significant changes in the mRNA abundance and the overlap between the normalized samples, eight mRNAs were found to be unique for LNCaP MVs, 17 were exclusive for PC-3 MVs, and only eight mRNAs were significantly abundant and common for MVs from both PCa cell lines (Fig. 4d and Additional file 2: Table S1, Additional file 3: Table S2 and Additional file 4: Table S3). This argues that different cancer cell lines from the same cancer type can release a vesicular secretome carrying fundamentally different mRNA transcripts.

Next, we determined which of the common mRNAs for both the PCa MVs had statistically significant different levels compared to the control PNT2 MVs (Table 1). PCa *vs* control comparisons revealed some startling increases of mRNA levels of: *ETV1*, a gene that directs androgen metabolism and confers aggressive PCa [23]; *FASN*, a fatty acid metabolism gene highly up-regulated in PCa [31]; *RBM39*, a gene implicated in colorectal and breast cancer progression [32, 33]. On the other hand, we also observed decreases in the mRNA levels of genes such as *CCND2*, a crucial cell cycle-regulatory gene down-regulated in PCa cells [34]. Interestingly, the levels of the transcription factor *ERG* whose up-regulation is a poor prognosis indicator [23], and *GNRH1*, *HAL*, and *LGALS4* genes were decreased in the PCa-derived MVs.

**Table 1:** Top Commonly Regulated Genes between LNCaP MVs and PC-3 MVs *vs* PNT2 MVs

				LNCaP MVs		PC-3 MVs	
Gene Symbol	Name	Unigene ID	RefSeq	Log FC	*P* Value	Log FC	*P* Value
Up-regulated							
ETV1	Ets variant 1	Hs.574240	NM_004956	4.3	0.042	3.4	0.02
FASN	Fatty acid synthase	Hs.83190	NM_004104	5.3	0.016	5.8	0.01
RBM39	RNA binding motif protein 39	Hs.282901	NM_004902	4.7	0.027	5.3	<0.001
Down-regulated							
CCND2	Cyclin D2	Hs.376071	NM_001759	−7.7	0.030	−6.2	0.031
ERG	V-ets erythroblastosis virus E26 oncogene homolog (avian)	Hs.473819	NM_182918	−4.3	0.049	−7.3	0.041
GNRH1	Gonadotropin-releasing hormone 1 (luteinizing-releasing hormone)	Hs.82963	NM_000825	−4.0	0.029	−4.5	0.024
HAL	Histidine ammonia-lyase	Hs.190783	NM_002108	−5.7	<0.001	−6.3	<0.001
LGALS4	Lectin, galactoside-binding, soluble, 4	Hs.5302	NM_006149	−4.1	0.034	−3.6	0.037

Log_2_ fold-change (mean of the differential mRNA levels); *P* value for the differential mRNA levels

### Differential expression of the mRNAs in other EVs, prostate cancer cell lines, and prostate biopsies

**Fig. 5:**
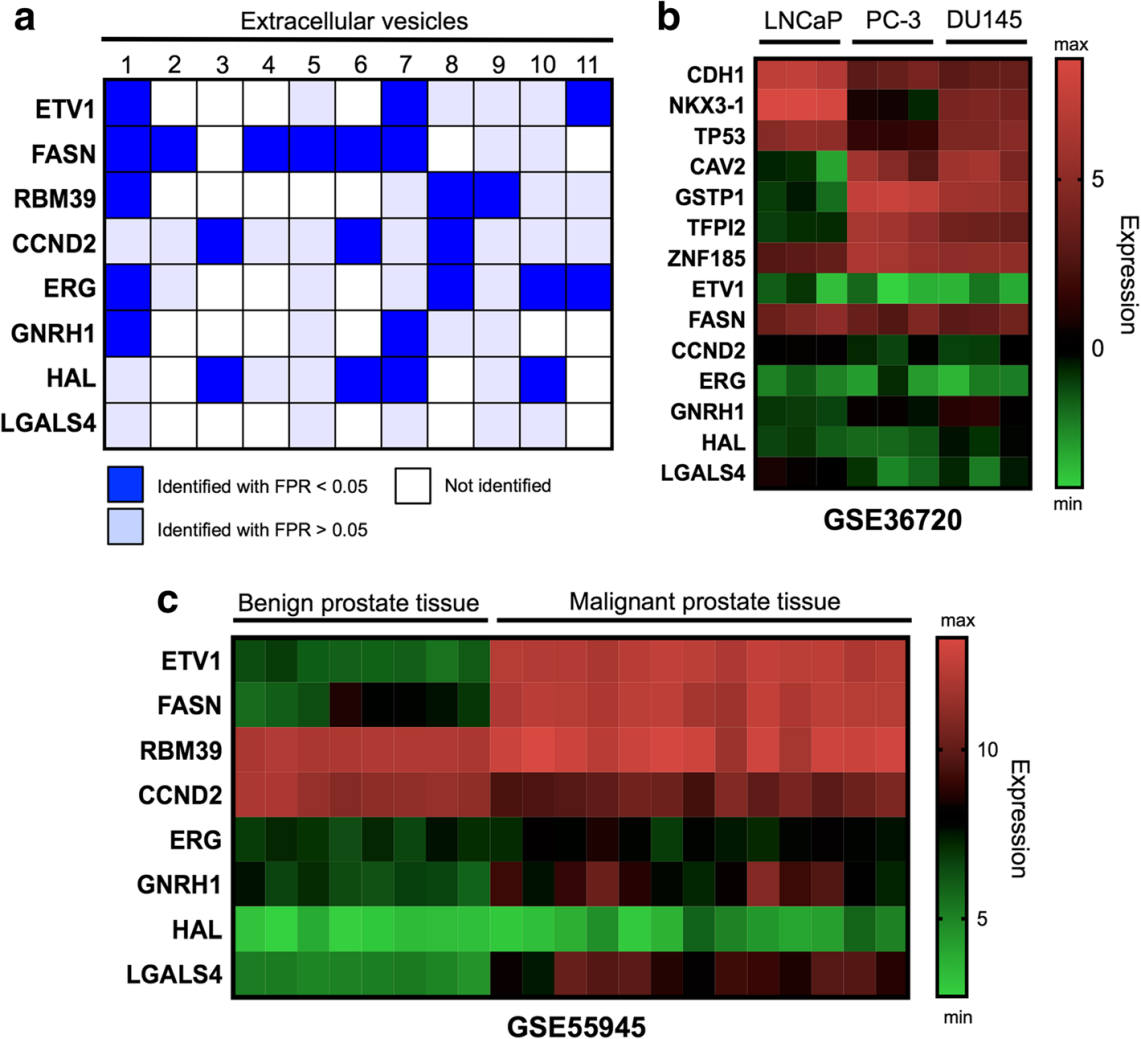
Comparative analysis of MV-mRNAs previously identified in other EVs, prostate cancer cell lines, and prostate benign and malignant tissues. **a** Heat map displaying the presence (*blue*) or absence (*white*) of the eight commonly identified mRNAs for the *ETV1*, *FASN*, *RBM39*, *CCND2*, *ERG*, *GNRH1*, *HAL* and *LGALS4* genes in eleven different microarray studies of mRNA content of EVs from EVpedia database. *blue* = identified with a false positive ration (FPR) <0.05; *light blue* = identified with a FPR >0.05; *white* = not identified. **b** Heat map showing the expression of 14 genes in LNCaP, PC-3 and DU-145 cell lines from a dataset with accession number GSE36720. **c** Heat map displaying the differential expression of the mRNAs of eight genes in malignant prostate tissue as compared to benign prostate tissue. Dataset with accession number GSE55945. Results between different microarray studies are not comparable

To evaluate the presence of the common PCa mRNAs identified in LNCaP and PC-3 MVs in EVs from different sources, a comparison was performed with all the studies collected in EVpedia were these mRNAs were identified in EVs [35] (Fig. 5a). A very limited number of reports (2–4 out of 11) identified the mRNAs in EVs from different cancer and non-cancerous cell sources, with the exception of *FASN*, which was found in 6/10 studies. Interestingly, *LGALS4* mRNA has been first identified and validated in this study. To further assess whether the statistically significant common mRNAs found in PCa MVs were cell-line specific and represented specific PCa signatures, we compared them with a publically available dataset (GSE36720) in the GEO database of mRNAs expressed in different PCa cell lines (Fig. 5b). *CDH1*, *NKX3-1*, and *TP53* genes were over-expressed in LNCaP cells compared with PC-3 cells. Contrary, *CAV2*, *GSTP1*, *TFPI2*, and *ZNF185* genes were over-expressed in PC-3 cells in comparison with LNCaP cells, and *ETV1*, *FASN*, *CCND2*, *ERG*, *GNRH1*, *HAL,* and *LGALS4* genes exhibited similar expression in both cell lines. Finally, as a proof of concept to evaluate the possible clinical relevance of the common mRNAs for both PC-3 and LNCaP MVs, the results were compared with a publically available microarray dataset in the GEO database containing information of mRNA expression on benign and malignant prostate tissue (GSE55945) (Fig. 5c). Here, some of the significant common mRNAs discovered in the PCa MVs, including *ETV1* and *FASN* genes, were significantly differentially expressed in the malignant prostate tissue in comparison with the benign tissue. This finding suggests that the mRNAs from *ETV1* and *FASN* genes found to be significantly enriched in MVs from LNCaP and PC-3 cells correlated with prostate cancer in patient samples, demonstrating the possible future applicability of the mRNA analysis using MVs.

## Discussion

In this study, we analyzed the mRNAs of 84 genes known to be involved in PCa from EV subpopulations derived from human PCa cell lines. LNCaP and PC-3 cells were chosen for EV isolation due to their extensive use as PCa cell models. To compare the relative abundance of mRNAs in EVs, the same subpopulations were isolated from PNT2 cells, a benign human prostatic epithelial cell line [36]. Differential centrifugation, the gold standard technique in the EV isolation [37], allowed the separation of enriched MVs and EXOs fractions, as previously reported [20, 38]. Despite its drawbacks, differential centrifugation is still the only technique that results in the separation of enriched, larger and smaller EV subpopulations albeit overlapping and non-homogeneous. PCa MVs and EXOs carried common EV proteins irrespective of being derived from malignant or non-malignant cells, and they had similar morphology and size range independent of their cellular origin, as described by our group [5, 6, 20, 38] and others [7, 39].

EVs from both normal and cancer cells can carry both intact and fragmented mRNA with a size range between 25 and 700 nt, which is considerably different from the intact cellular mRNAs, sized between 400 nt to 12,000 nt [40–42]. The presence of 18S and 28S rRNA in EVs has been reported rather ambiguously. In our study, and consistent with what others have previously published, MVs contained significantly more rRNA than EXOs, which were primarily enriched in smaller RNAs of < 700 nt, despite the presence of larger RNA fragments [5–7]. However, the PNT2-derived EXOs had a bigger proportion of rRNA than the EXOs from PC-3 and LNCaP cells, which may imply that specific RNA sorting signals and mechanisms exist for distinct subtypes of EVs or originator cells [14, 16, 43–46].

RNA signatures of EVs can be specific for the cells of their origin [36, 42] discriminating e.g. the cancerous transformation status of cells. In this study, MVs were found to be significantly enriched in several of the mRNAs analyzed when compared to EXOs. Based on these results, MVs rather than EXOs might better represent a population of vesicles with an advantage for translational studies. Since MVs are larger vesicles, they might also contain more tumor-derived molecules including metabolites, proteins, and nucleic acids. Moreover, the RNA profile of MVs seems to be more similar to the donor cells than that of EXOs [5, 6], thereby giving a snapshot of the tumor transcriptome. This is an important finding, since the emphasis of EVs in cancer research has so far heavily been on EXOs and only a few studies have highlighted the advantages of larger vesicles, including large oncosomes [47]. However, more research is needed in order to elucidate whether tumor cells modulate the EV cargo at different stages of the disease and how the specific sorting of molecules into EVs takes place.

Here, the relative abundance of mRNAs was analyzed for different types of genes associated with PCa: differentially methylated promoters, androgen-signaling pathway, PI3 kinase/AKT and PTEN signaling pathways, apoptosis, cell cycle, and metastatic potential. Among them, statistical significant mRNA levels of 16 genes were at least 10-fold differentially regulated in the LNCaP MVs and 32 in PC-3 MVs when compared to the control PNT2 MVs. Interestingly, mRNAs sequences of only three genes were uniquely increased in LNCaP MVs in comparison with PNT2 MVs. These included the *NKX3-1* gene, which is one of the earliest markers for prostate development during embryogenesis and is a regulator of cell proliferation, differentiation, and apoptosis [23]; the *TP53* gene, which is well-known for tumor recurrence and metastasis [48], and the androgen-regulated *TMPRSS2 * gene, which is highly expressed in PCa [49, 50]. In contrast, eight mRNAs were exclusively present in PC-3 MVs, some of them include *CAV2* gene, a cell cycle regulator involved in PCa progression [27]; the *GSTP1* gene, whose hypermethylation is one of the most frequently observed aberrations in PCa [28]; *PES1*, *CAMSAP1,* and *CCND1* genes, up-regulated or correlated with PCa development [51–53] and the *ZNF185* gene, which is frequently reported to be dysregulated in PCa [54]. Overall, the unique mRNA signature of LNCaP and PC-3 MVs allows the discrimination of the characteristics of their parental cells, such as androgen sensitivity or metastatic potential, since MVs represent their source of cell origin. The common mRNAs in MVs were first identified and validated in PCa EVs in our study, and very limited number of reports previously identified those mRNAs in other cancer-derived EVs [35]. Furthermore, another interesting finding was the possible clinical relevance of *ETV1* and *FASN* mRNA transcripts. As determined by cluster analysis, *ETV1* and *FASN* were differential over-expressed in malignant prostate tissue in comparison with benign biopsies, highlighting their possible relevance as future cancer markers.

The majority of the transcriptomic reports studying EVs utilize microarray analyses and deep sequencing to simultaneously examine the expression of thousands of genes [6, 15–17]. While these technologies represent a great opportunity for clinical research, the results need further validation [55, 56]. To our knowledge, this is the first study showing and validating the changes in mRNA levels of 84 genes associated with PCa by RT-qPCR in different subsets of PCa cell-derived EVs. The presented results provide important leads to identify specific markers in MVs and EXOs isolated from both cancer cells and biofluids of cancer patients, which could have a utility for cancer diagnosis and response monitoring of cancer treatments.

## Conclusions

The mRNA cargo in EVs considerably differs based on the EV subpopulation analyzed and the EV cellular origin, as different PCa cells and benign prostate epithelial cells release very different mRNAs in their respective EVs. The mRNA analyses performed in this study therefore provide new insights into the proportion of the cell transcriptome that can be detected within EVs. The data also emphasize the need to better dissect the role of EVs and their cargo in cancer cell phenotype, putatively in relation to progression of disease. Since the molecular cancer signatures can be identified in EVs, their analysis can contribute to the understanding of differences in PCa heterogeneity. Whilst many previous studies have focused on EV-associated miRNAs, this work demonstrates the potential of the selective analysis of the mRNA content of MVs and EXOs and highlights their applicability in biomarker research.

## Additional files

Additional file 1: Figure S1. RNA analysis of EV subpopulations by RT-qPCR. (A) Bar graph representing the threshold cycle (Ct) value range for the percentage of genes detected in LNCaP-, PC-3- and PNT2- MVs and EXOs. Higher Ct values represent lower mRNA enrichment. Bars represent the mean ± SEM of three independent experiments per group. (B-D) Individual Ct values representation for the 84 mRNA transcripts detected in LNCaP-, PC-3- and PNT2- MVs and EXOs. (PDF 133 kb)
Additional file 2: Table S1. Top Regulated Genes: Distinctly different genes between LNCaP MVs vs PNT2 MVs. (XLSX 9 kb)
Additional file 3: Table S2. Top Regulated Genes: Distinctly different genes between PC-3 MVs vs PNT2 MVs. (XLSX 10 kb)
Additional file 4: Table S3. Top Regulated Genes: Commonly regulated genes between LNCaP MVs vs PC-3 MVs. (XLSX 11 kb)

## Abbreviations

EVs: Extracellular vesicles
EXOs: Exosomes
mRNA: messenger RNA
MVs: Microvesicles
PCa: Prostate cancer
RT-qPCR: Quantitative reverse transcription PCR.

## References

[1] Mittelbrunn M, Sanchez-Madrid F (2012). Intercellular communication: diverse structures for exchange of genetic information. Nat Rev Mol Cell Biol.

[2] Yanez-Mo M (2015). Biological properties of extracellular vesicles and their physiological functions. J Extracell Vesicles.

[3] Meehan K, Vella LJ (2016). The contribution of tumour-derived exosomes to the hallmarks of cancer. Crit Rev Clin Lab Sci.

[4] Kanada M (2015). Differential fates of biomolecules delivered to target cells via extracellular vesicles. Proc Natl Acad Sci U S A.

[5] Crescitelli R, et al. Distinct RNA profiles in subpopulations of extracellular vesicles: apoptotic bodies, microvesicles and exosomes. J Extracell Vesicles. 2013;2:20677. 10.3402/jev.v2i0.20677 PMC3823106 24223256

[6] Lunavat TR (2015). Small RNA deep sequencing discriminates subsets of extracellular vesicles released by melanoma cells - Evidence of unique microRNA cargos. RNA Biol.

[7] Willms E (2016). Cells release subpopulations of exosomes with distinct molecular and biological properties. Sci Rep.

[8] Raposo G, Stoorvogel W (2013). Extracellular vesicles: exosomes, microvesicles, and friends. J Cell Biol.

[9] Valadi H (2007). Exosome-mediated transfer of mRNAs and microRNAs is a novel mechanism of genetic exchange between cells. Nat Cell Biol.

[10] Ratajczak J (2006). Embryonic stem cell-derived microvesicles reprogram hematopoietic progenitors: evidence for horizontal transfer of mRNA and protein delivery. Leukemia.

[11] Montecalvo A (2012). Mechanism of transfer of functional microRNAs between mouse dendritic cells via exosomes. Blood.

[12] Alvarez-Erviti L (2011). Delivery of siRNA to the mouse brain by systemic injection of targeted exosomes. Nat Biotechnol.

[13] Ekstrom K, et al. Characterization of mRNA and microRNA in human mast cell-derived exosomes and their transfer to other mast cells and blood CD34 progenitor cells. J Extracell Vesicles. 2012;1:18389. 10.3402/jev.v1i0.18389 PMC3760639 24009880

[14] Skog J (2008). Glioblastoma microvesicles transport RNA and proteins that promote tumour growth and provide diagnostic biomarkers. Nat Cell Biol.

[15] Nolte-'t Hoen EN (2012). Deep sequencing of RNA from immune cell-derived vesicles uncovers the selective incorporation of small non-coding RNA biotypes with potential regulatory functions. Nucleic Acids Res.

[16] Bellingham SA, Coleman BM, Hill AF (2012). Small RNA deep sequencing reveals a distinct miRNA signature released in exosomes from prion-infected neuronal cells. Nucleic Acids Res.

[17] Vojtech L (2014). Exosomes in human semen carry a distinctive repertoire of small non-coding RNAs with potential regulatory functions. Nucleic Acids Res.

[18] Aatonen MT, et al. Isolation and characterization of platelet-derived extracellular vesicles. J Extracell Vesicles. 2014;3:24692. 10.3402/jev.v3.24692 PMC4125723 25147646

[19] Dong L (2016). Circulating Long RNAs in Serum Extracellular Vesicles: Their Characterization and Potential Application as Biomarkers for Diagnosis of Colorectal Cancer. Cancer Epidemiol Biomarkers Prev.

[20] Lazaro-Ibanez E (2014). Different gDNA content in the subpopulations of prostate cancer extracellular vesicles: apoptotic bodies, microvesicles, and exosomes. Prostate.

[21] Livak KJ, Schmittgen TD (2001). Analysis of relative gene expression data using real-time quantitative PCR and the 2(-Delta Delta C(T)) Method. Methods.

[22] LeBeau AM (2010). Prostate-specific antigen: an overlooked candidate for the targeted treatment and selective imaging of prostate cancer. Biol Chem.

[23] Shen MM, Abate-Shen C (2010). Molecular genetics of prostate cancer: new prospects for old challenges. Genes Dev.

[24] Shinojima T (2012). Heterogeneous epigenetic regulation of TIMP3 in prostate cancer. Epigenetics.

[25] Yang G (1998). Elevated expression of caveolin is associated with prostate and breast cancer. Clin Cancer Res.

[26] Yang G (1999). Caveolin-1 expression in clinically confined human prostate cancer: a novel prognostic marker. Cancer Res.

[27] Sugie S (2015). Significant Association of Caveolin-1 and Caveolin-2 with Prostate Cancer Progression. Cancer Genomics Proteomics.

[28] Meiers I, Shanks JH, Bostwick DG (2007). Glutathione S-transferase pi (GSTP1) hypermethylation in prostate cancer: review 2007. Pathology.

[29] Ameri A (2011). Prognostic Value of Promoter Hypermethylation of Retinoic Acid Receptor Beta (RARB) and CDKN2 (p16/MTS1) in Prostate Cancer. Chin J Cancer Res.

[30] Konduri SD (2001). Overexpression of tissue factor pathway inhibitor-2 (TFPI-2), decreases the invasiveness of prostate cancer cells in vitro. Int J Oncol.

[31] Huang M (2016). Diet-induced alteration of fatty acid synthase in prostate cancer progression. Oncogenesis.

[32] Mercier I (2014). CAPER, a novel regulator of human breast cancer progression. Cell Cycle.

[33] Sillars-Hardebol AH (2012). CSE1L, DIDO1 and RBM39 in colorectal adenoma to carcinoma progression. Cell Oncol (Dordr).

[34] Henrique R (2006). Hypermethylation of Cyclin D2 is associated with loss of mRNA expression and tumor development in prostate cancer. J Mol Med (Berl).

[35] Kim DK (2015). EVpedia: a community web portal for extracellular vesicles research. Bioinformatics.

[36] Ahadi A (2016). Long non-coding RNAs harboring miRNA seed regions are enriched in prostate cancer exosomes. Sci Rep.

[37] Thery C (2006). Isolation and characterization of exosomes from cell culture supernatants and biological fluids. Curr Protoc Cell Biol.

[38] Saari H (2015). Microvesicle- and exosome-mediated drug delivery enhances the cytotoxicity of Paclitaxel in autologous prostate cancer cells. J Control Release.

[39] Osteikoetxea X (2015). Improved characterization of EV preparations based on protein to lipid ratio and lipid properties. PLoS One.

[40] Batagov AO, Kurochkin IV (2013). Exosomes secreted by human cells transport largely mRNA fragments that are enriched in the 3'-untranslated regions. Biol Direct.

[41] Enderle D (2015). Characterization of RNA from Exosomes and Other Extracellular Vesicles Isolated by a Novel Spin Column-Based Method. PLoS One.

[42] Jenjaroenpun P (2013). Characterization of RNA in exosomes secreted by human breast cancer cell lines using next-generation sequencing. PeerJ.

[43] Hessvik NP (2012). Profiling of microRNAs in exosomes released from PC-3 prostate cancer cells. Biochim Biophys Acta.

[44] Mittelbrunn M (2011). Unidirectional transfer of microRNA-loaded exosomes from T cells to antigen-presenting cells. Nat Commun.

[45] Pigati L (2010). Selective release of microRNA species from normal and malignant mammary epithelial cells. PLoS One.

[46] Rabinowits G (2009). Exosomal microRNA: a diagnostic marker for lung cancer. Clin Lung Cancer.

[47] Minciacchi VR (2015). Large oncosomes contain distinct protein cargo and represent a separate functional class of tumor-derived extracellular vesicles. Oncotarget.

[48] Chen SL (2003). P53 is a regulator of the metastasis suppressor gene Nm23-H1. Mol Carcinog.

[49] Lin B (1999). Prostate-localized and androgen-regulated expression of the membrane-bound serine protease TMPRSS2. Cancer Res.

[50] Vaarala MH (2001). The TMPRSS2 gene encoding transmembrane serine protease is overexpressed in a majority of prostate cancer patients: detection of mutated TMPRSS2 form in a case of aggressive disease. Int J Cancer.

[51] Chandran UR (2007). Gene expression profiles of prostate cancer reveal involvement of multiple molecular pathways in the metastatic process. BMC Cancer.

[52] Nakamura Y (2013). Cyclin D1 (CCND1) expression is involved in estrogen receptor beta (ERbeta) in human prostate cancer. Prostate.

[53] Li Y, Sarkar FH (2002). Gene expression profiles of genistein-treated PC3 prostate cancer cells. J Nutr.

[54] Zhang JS, Gong A, Young CY (2007). ZNF185, an actin-cytoskeleton-associated growth inhibitory LIM protein in prostate cancer. Oncogene.

[55] Koshiol J (2010). No role for human papillomavirus in esophageal squamous cell carcinoma in China. Int J Cancer.

[56] Abdullah-Sayani A, Bueno-de-Mesquita JM, van de Vijver MJ (2006). Technology Insight: tuning into the genetic orchestra using microarrays--limitations of DNA microarrays in clinical practice. Nat Clin Pract Oncol.

