# Supplementary material for: Distinct prostate cancer-related mRNA cargo in extracellular vesicle subsets from prostate cell lines
Source: BMC Cancer. 2017 Feb 1;17:92. doi: 10.1186/s12885-017-3087-x (PMC5286827; doi:10.1186/s12885-017-3087-x)
Supplement: Additional file 1: Figure S1. — RNA analysis of EV subpopulations by RT-qPCR. (A) Bar graph representing the threshold cycle (Ct) value range for the percentage of genes detected in LNCaP-, PC-3- and PNT2- MVs and EXOs. Higher Ct values represent lower mRNA enrichment. Bars represent the mean ± SEM of three independent experiments per group. (B-D) Individual Ct values representation for the 84 mRNA transcripts detected in LNCaP-, PC-3- and PNT2- MVs and EXOs. (PDF 133 kb) [file 12885_2017_3087_MOESM1_ESM.pdf]

Additional file 1
Supplementary figure

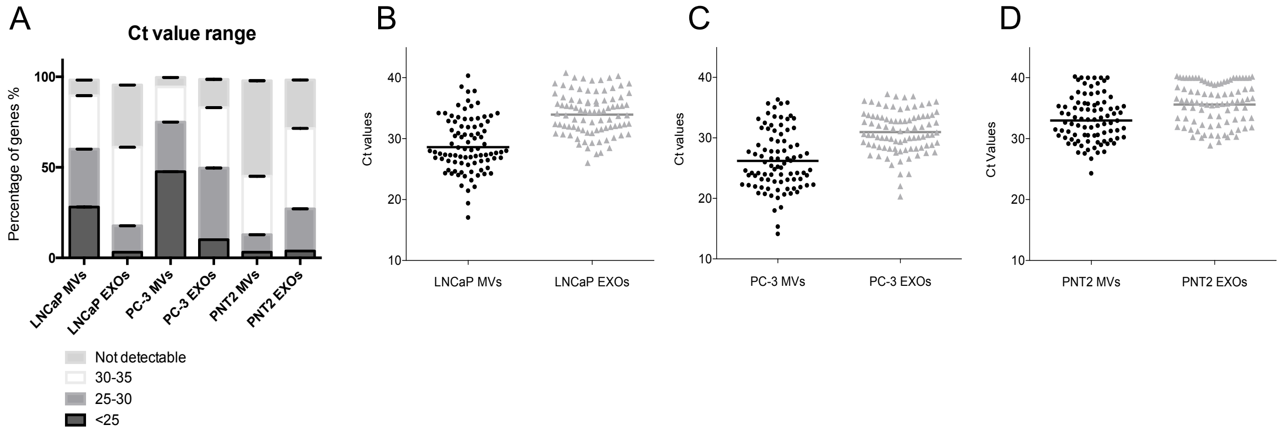

Figure 1S. RNA analysis of EV subpopulations by RT-qPCR.

(**A**) Bar graph representing the threshold cycle (Ct) value range for the percentage of genes detected in LNCaP-, PC-3- and PNT2- MVs and EXOs. Higher Ct values represent lower mRNA enrichment. Bars represent the mean ± SEM of three independent experiments per group. (**B**-**D**) Individual Ct values representation for the 84 mRNA transcripts detected in LNCaP-, PC-3- and PNT2- MVs and EXOs.
